# Tuning of Hemes *b* Equilibrium Redox Potential Is Not Required for Cross-Membrane Electron Transfer*

**DOI:** 10.1074/jbc.M115.712307

**Published:** 2016-02-08

**Authors:** Sebastian Pintscher, Patryk Kuleta, Ewelina Cieluch, Arkadiusz Borek, Marcin Sarewicz, Artur Osyczka

**Affiliations:** From the Department of Molecular Biophysics, Faculty of Biochemistry, Biophysics and Biotechnology, Jagiellonian University, 30-387 Kraków, Poland

**Keywords:** cytochrome, electron transfer, oxidation-reduction (redox), photosynthesis, respiratory chain, heme iron ligation

## Abstract

In biological energy conversion, cross-membrane electron transfer often involves an assembly of two hemes *b*. The hemes display a large difference in redox midpoint potentials (Δ*E*_m__b), which in several proteins is assumed to facilitate cross-membrane electron transfer and overcome a barrier of membrane potential. Here we challenge this assumption reporting on heme *b* ligand mutants of cytochrome *bc*_1_ in which, for the first time in transmembrane cytochrome, one natural histidine has been replaced by lysine without loss of the native low spin type of heme iron. With these mutants we show that Δ*E*_m__b can be markedly increased, and the redox potential of one of the hemes can stay above the level of quinone pool, or Δ*E*_m__b can be markedly decreased to the point that two hemes are almost isopotential, yet the enzyme retains catalytically competent electron transfer between quinone binding sites and remains functional *in vivo*. This reveals that cytochrome *bc*_1_ can accommodate large changes in Δ*E*_m__b without hampering catalysis, as long as these changes do not impose overly endergonic steps on downhill electron transfer from substrate to product. We propose that hemes *b* in this cytochrome and in other membranous cytochromes *b* act as electronic connectors for the catalytic sites with no fine tuning in Δ*E*_m__b required for efficient cross-membrane electron transfer. We link this concept with a natural flexibility in occurrence of several thermodynamic configurations of the direction of electron flow and the direction of the gradient of potential in relation to the vector of the electric membrane potential.

## Introduction

Redox midpoint potential (*E*_m_) is a key property of any redox active cofactor in proteins. It regulates biological functions via thermodynamic and kinetic control of electron exchange reactions. Because these reactions must take place in a variety of cellular compartments, both outside and inside the biological membrane, the structures of redox proteins have evolved to meet physicochemical requirements of these various environments to achieve assemblies that secure functionally competent *E*_m_ values.

Within the group of cytochromes, molecular factors that modulate *E*_m_ include types of heme axial ligation (1–3). The residues that are most commonly recruited as axial ligands for the heme iron are His and/or Met (4). A binding of hemes *b* within the membranous proteins is accomplished by an assembly of transmembrane α-helices that provide His axial ligands for the heme-iron. In fact, the heme binding α-helix bundle represents a common motif of several bioenergetic complexes (5–7). It has even been used as a prototype to construct human-made versions of heme binding proteins (protein maquettes) (8–10).

**FIGURE 1.**
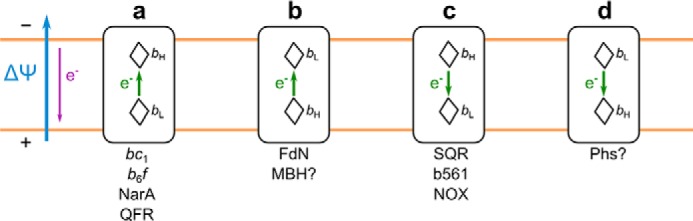
**Possible thermodynamic configurations for cross-membrane electron transfer in cytochromes *b*.** *a*, electron is transferred against ΔΨ and involves energetically favorable reduction of heme *b*_H_ by heme *b*_L_. *b*, electron is transferred against ΔΨ and additionally involves energetically unfavorable reduction of heme *b*_L_ by heme *b*_H_. *c*, energetically unfavorable reduction of heme *b*_L_ by heme *b*_H_ is facilitated by ΔΨ. *d*, energetically favorable reduction of heme *b*_H_ by heme *b*_L_ is additionally facilitated by ΔΨ. *b*_L_ and *b*_H_ denote low potential and high potential heme, respectively. The *blue arrow* refers to ΔΨ (membrane electric potential); the *purple arrow* indicates the direction of ΔΨ-induced electron transfer; and the *green arrows* indicate direction of electron transfer between hemes. Configuration *a* can be found in cytochrome *bc*_1_ (11, 12), cytochrome *b*_6_*f* (37), nitrate reductase A (*NarA*) (39, 40), and fumarate reductase (*QFR*) (41, 42); configuration *b* can be found in formate dehydrogenase N (*FdN*) (40, 43) and perhaps in membrane-bound [Ni-Fe] hydrogenase (*MBH*) (45, 46); configuration *c* can be found in succinate dehydrogenase (*SQR*) (47, 48), cytochromes *b*_561_ (*b561*) (52–54), and NADPH oxidase (*NOX*) (51); and configuration *d* probably exists in thiosulfate reductase (*Phs*) (55).

The α-helix bundle can bind one or two hemes *b*. In several proteins, an assembly of two *b* type hemes, each facing different sides of the membrane, supports electron transfer across biological membranes crucial for energy conservation in many systems. Intriguingly, the two hemes differ largely in their redox midpoint potentials (the *E*_m_ difference, Δ*E*_m__b, is typically in the range of 100 mV); however, the thermodynamic rationale behind the existence of Δ*E*_m__b remains unclear. This is because no general rule for the direction of Δ*E*_m__b with respect to the direction of the electric field generated by the membrane potential or the direction of physiological electron transfer is evident (Fig. 1).

The cytochrome *b* subunit of cytochrome *bc*_1_ (mitochondrial complex III) is a well known example of a protein supporting cross-membrane electron transfer by using an assembly of two hemes *b*, named heme *b*_H_ and heme *b*_L_, where subscripts H and L refer to high and low potential, respectively (for recent reviews see Refs. 11 and 12). During the catalytic cycle, the electron transfer from heme *b*_L_ to heme *b*_H_ connects the quinol oxidation site (Q_o_)^2^ and the quinone reduction site (Q_i_). In addition, in dimeric structure of an enzyme, intermonomer electron transfer parallel to the membrane plane involving two hemes *b*_L_ is possible (13–16). In living cells, the cross-membrane electron flow from heme *b*_L_ to heme *b*_H_ may face the barrier of the membrane potential (Fig. 1*a*). Thus, the fact that electrons are transferred from the cofactor of lower *E*_m_ to the cofactor of higher *E*_m_ provided a basis for a general assumption that Δ*E*_m__b is one of the factors that facilitate cross-membrane electron transfer and perhaps is important in overcoming the barrier of potential (17, 18). However, the contribution of Δ*E*_m__b to the overall electron flow has not been verified experimentally. Prerequisites for such verification are variants of cytochrome *bc*_1_ with large changes in the *E*_m_ of hemes *b* and, consequently, large changes in Δ*E*_m__b. However, the mutations of cytochrome *b* tested so far either had a relatively small effect on the *E*_m_ of hemes *b* (19, 20) or resulted in the absence of the heme (21, 22).

Here we mutated the native bis-His coordination pattern for heme *b*_L_ and/or heme *b*_H_ into the His-Lys pattern, to our knowledge, providing the first His-Lys coordinated hemes *b* in a transmembrane protein. The hemes remain low spin as in a native enzyme but have markedly elevated *E*_m_ values and thus effectively modulate Δ*E*_m__b: an increase in the *E*_m_ of heme *b*_H_ by 50 mV increased Δ*E*_m__b setting the *E*_m_ of heme *b*_H_ above the *E*_m_ of the quinone pool in the membrane, whereas an increase in the *E*_m_ of heme *b*_L_ by 155 mV decreased Δ*E*_m__b to the point that the two hemes *b* were almost isopotential. This provided an unprecedented set of large changes in Δ*E*_m__b for functional testing. The results offer a new perspective toward understanding the natural engineering of the cross-membrane electron transfer in cytochromes *b*.

## Experimental Procedures

### 

#### 

##### Bacterial Strains, Plasmids, and Growth Conditions

*Rhodobacter capsulatus* and *Escherichia coli* (HB101, DH5α) were grown in liquid or solid MPYE (mineral-peptone-yeast extract) and LB (Luria Bertani) media, at 30 and 37 °C, respectively, supplemented with appropriate antibiotics as needed. Respiratory growth of *R. capsulatus* strains was achieved at 30 °C in the dark under semiaerobic conditions. Photosynthetic growth abilities of mutants were tested on MPYE plates using anaerobic jars (GasPak^TM^ EZ Anaerobe Container System; BD Biosciences) at 30 °C under continuous light. The *R. capsulatus* strains used were: pMTS1/MTR*bc*_1_ which overproduces wild-type cytochrome *bc*_1_ from the expression vector pMTS1 (contains a copy of pet*ABC* operon coding for all three subunits of cytochrome *bc*_1_), and MTR*bc*_1_ which is a pet*ABC* operon deletion background (23). The mutagenized pMTS1 derivatives were introduced to *R. capsulatus* MTR*bc*_1_ via triparental crosses as described (23). Plasmid pPET1 (a derivative of pBR322 containing a wild-type copy of pet*ABC*) was used as a template for PCR and in some of the subcloning procedures.

##### Construction of Lys Mutants

Spontaneous Ps^+^ revertant of the H212N mutant originally described in Ref. 22 was obtained on MPYE plate containing tetracycline after ∼7 days of cultivation under photosynthetic conditions. The DNA sequence analysis of the plasmid DNA isolated from the revertant strain revealed a single base pair change replacing the mutated Asn into Lys at position 212 of cytochrome *b*. The XmaI/SfuI fragment containing the reversion (mutation H212K), and no other mutations was exchanged with its counterpart on expression vector pMTS1 carrying the wild-type copy of the pet*ABC* operon. Expression of this vector in the MTR*bc*_1_ background strain confirmed that cells bearing single mutation H212K display the photosynthetically competent Ps^+^ phenotype. The Ps^+^ reversion H212K occurred also in the double mutant A181T/H212N cultivated on MPYE plate containing kanamycin (in this case H212N in the cytochrome *b* subunit was accompanied by a mutation A181T in cytochrome *c*_1_ described originally in Ref. (24)). Because A181T/H212N, unlike original H212N, contained cytochrome *b* already equipped with the Strep-tag II attached to its carboxyl end, the plasmid obtained from the revertant of A181T/H212N was used to construct the mutants used in further analysis. First, the XmaI/SfuI fragment containing the reversion (H212K) and the sequence coding for Strep-tag II (ST), and no other mutations were exchanged with its counterpart on expression vector pMTS1 carrying the wild-type copy of the pet*ABC* operon. This created pMTS1-ST-H212K. Second, the same XmaI/SfuI fragment was cloned into pPET1 creating pPET1-ST-H212K. Mutation H198K and the double mutation H212K/H198K were constructed by the QuikChange site-directed mutagenesis kit from Stratagene using pPET1-ST (25) and pPET1-ST-H212K plasmids as templates, respectively, and the mutagenic forward H198K-F (5′-GGGCAGCAGATATTTCAGCGAGAAGAAGCGG-3′) and reverse H198K-R (5′-TTCTTCTCGCTGAAATATCTGCTGCCCTTCG-3′) oligonucleotides. After sequencing, XmaI/SfuI fragments of pPET1 plasmids bearing the desired mutations, and no other mutations were exchanged with their wild-type counterparts in pMTS1. This created the plasmids pMTS1-ST-H198K and pMTS1-ST-H212K/H198K. Plasmids pMTS1-ST-H212K, pMTS1-ST-H198K, and pMTS1-ST-H212K/H198K were inserted into *R. capsulatus* MTRBC1 cells, creating mutants H212K, H198K, and H212K/H198K, respectively. These mutants are listed in Table 1. In each case, the presence of introduced mutations was confirmed by sequencing the plasmid DNA reisolated from the mutated *R. capsulatus* strains.

##### Isolation of Membranes and Proteins

Chromatophore membranes were isolated from *R. capsulatus* as described previously (26). The cytochrome *bc*_1_ complexes were isolated from detergent-solubilized chromatophores by affinity chromatography using the procedure described previously (27). SDS-PAGE of purified complexes was performed as described before (28).

##### Optical and EPR Spectroscopy

Optical spectra measurements of isolated complexes and determination of protein concentration were performed on UV-2450 Shimadzu spectrophotometer. Cytochrome *bc*_1_ samples were suspended in 50 mm Tris, pH 8.0, 100 mm NaCl, 0.01% (m/m) dodecyl maltoside, and 1 mm EDTA, an appropriate amount of ferricyanide was added to fully oxidize complexes; then solid ascorbate and solid sodium dithionite were added to reduce samples, and spectra were recorded right after oxidation and after each step of reduction. Concentration of cytochrome *bc*_1_ was determined as described (26). EPR measurements were performed on Bruker Elexys E580 spectrometer. X-band CW-EPR spectra of hemes were measured at 10 K, using SHQE0511 resonator combined with ESR900 Oxford Instruments cryostat unit, using 1.595 mT modulation amplitude, and 1.543 milliwatt of microwave power. For EPR measurements, cytochrome *bc*_1_ samples were dialyzed against 50 mm Tris, pH 8.0, 100 mm NaCl, 20% glycerol (v/v), 0.01% (m/m) dodecyl maltoside, and 1 mm EDTA. Final concentration of cytochrome *bc*_1_ in EPR samples was 50 μm. Antimycin A was used in 5-fold molar excess over the concentration of cytochrome *bc*_1_.

##### Redox Potentiometry

Midpoint potentials of hemes *b* were determined by dark equilibrium redox titrations on chromatophores according to the method described in Ref. (29). Chromatophores were suspended in argon-equilibrated 50 mm MOPS buffer (pH 7.0) containing 100 mm KCl and 1 mm EDTA. Immediately before the titration, the following redox mediators were added: 100 μm tetrachlorohydroquinone, 100 μm 2,3,5,6-tetramethyl phenylenediamine (*E*_m7_ 260 mV), 100 μm 1,2-naphtoquinone-4-sulfonate (*E*_m7_ 210 mV), 100 μm 1,2-naphtoquinone (*E*_m7_ 130 mV), 50 μm phenazine methosulfate (*E*_m7_ 80 mV), 50 μm phenazine ethosulfate (*E*_m7_ 50 mV), 100 μm duroquinone (*E*_m7_ 5mV), 30 μm indigotrisulfonate (*E*_m7_ = −90 mV), 100 μm 2-hydroxy-1,4-napthoquinone (*E*_m7_ = −152 mV), 100 μm anthroquinone-2-sulfonate (*E*_m7_ = −225 mV), and 100 μm benzyl viologen (*E*_m7_ = −374 mV). Dithionite and ferricyanide were used to adjust ambient redox potential. During the titrations, samples of 150–200 μl were taken and transferred to EPR tubes anaerobically and frozen by immersion into cold ethanol. The *E*_m7_ values of hemes *b* were determined by fitting the amplitudes of appropriate EPR *g*_z_ transitions to the Nernst equation for one-electron couple (for WT, H212K, and H198K) or for two one-electron couples (in case of H212K/H198K mutant).

##### Flash-induced Electron Transfer Measurements

Measurements of flash-induced turnover kinetics of cytochrome *bc*_1_ were performed on a home-built double wavelength time-resolved spectrophotometer as described in previous work (24). Chromatophores for measurements were suspended in 50 mm MOPS, pH 7.0, 100 mm KCl, 3.5 μm valinomycin, and redox mediators: 7 μm 2,3,5,6-tetramethyl-1,4-phenylenediamine, 1 μm phenazine methosulfate, 1 μm phenazine ethosulfate, 5.5 μm 1,2-naphthoquinone, and 5.5 μm 2-hydroxy-1,4-naphthoquinone (valinomycin and redox mediators were added immediately before the measurement). Dithionite and ferricyanide were used to adjust ambient redox potential. Inhibitors antimycin A and myxothiazol were used at final concentration of 7 μm. Transient hemes *b* reduction kinetics were followed at 560–570 nm (for WT and H198K) or 557–570 nm (for H212K and H212K/H198K). Transient hemes *c* oxidation and rereduction kinetics were followed at 550–540 nm. Single flash activation measurements were initiated by a short saturating flash (10 μs) from a xenon lamp, and multiple flash activation measurements were initiated by a series of short (10 μs) saturating flashes every 20 ms. In order to measure kinetics in the presence of the membrane potential, valinomycin was omitted. Rates of flash-induced heme *b* reduction were determined by fitting transient kinetics data to a single exponential equation.

##### Steady-state Kinetics Measurements

Steady-state enzymatic activities of cytochrome *bc*_1_ complexes in chromatophores were determined spectroscopically by the decylubiquinol-dependent reduction of bovine heart cytochrome *c* (Sigma-Aldrich) as described before (23). Conditions used in assays were as follows: 50 mm Tris-HCl (pH 8), 100 mm NaCl, 20 μm decylubiquinol, 20 μm oxidized cytochrome *c*. Errors were calculated as standard deviation of the mean of nine measurements. Chromatophores were treated with KCN (final concentration in sample was 0.5 mm) before experiments. Decylubiquinol was obtained as described (30).

## Results

### 

#### 

##### His-Lys Ligation for Hemes b in a Low Spin State Is Possible in Transmembrane Cytochrome b

**FIGURE 2.**
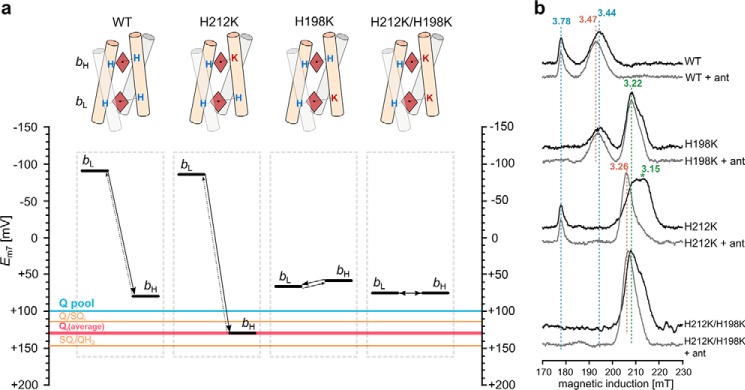
**His-Lys coordinated hemes *b* contain low spin heme iron and have markedly increased *E*_m_.** *a*, schematic representation of four-helix bundles (*tubes*) binding two hemes *b* (*brown diamonds*) and introduced changes in the ligation pattern; *H* and *K* denote His and Lys ligands of heme iron, respectively. The respective *E*_m_ diagrams are shown *below* the schemes. *Black bars* refer to *E*_m7_ of hemes, the *blue line* indicates *E*_m7_ of Q pool, *orange lines* indicate calculated *E*_m7_s of Q_i_/SQ_i_ and SQ_i_/Q_i_ couples (*upper* and *lower line*, respectively), and the *red line* indicates the resulting average *E*_m7_ of Q at the Q_i_ catalytic site. *b*, EPR spectra of hemes *b* in isolated complexes in the absence of inhibitors and presence of antimycin (*black* and *gray*, respectively). The *numbers* above the spectra and *dotted lines* denote values and positions of *g*_z_ transitions.

Changes in the coordination pattern of heme iron are expected to exert a large influence on the redox properties of hemes (1). Thus, to impose large shifts in *E*_m_ values of hemes *b* in cytochrome *b*, we created three variants in which one of the His ligands to heme iron was replaced by Lys (Lys mutants): single mutants H212K (for heme *b*_H_) and H198K (for heme *b*_L_) and the double mutant H212K/H198K combining both single mutations (Fig. 2*a*).

**FIGURE 3.**
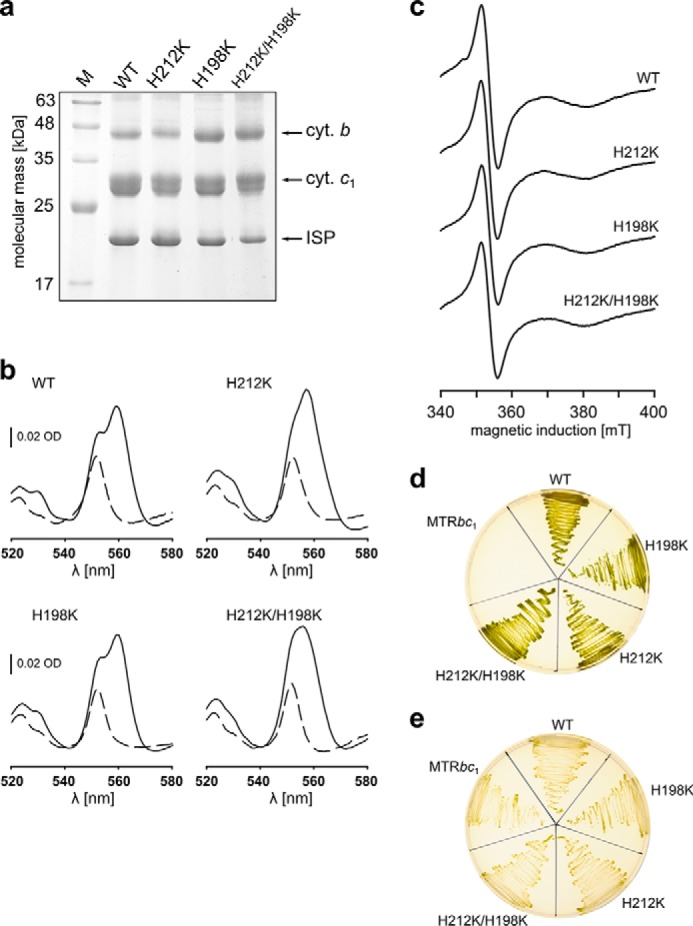
**SDS-PAGE profiles, spectral analyses, and growth capability of Lys mutants of cytochrome *bc*_1_.** *a*, Coomassie Blue-stained SDS-PAGE profiles of complexes isolated from solubilized membranes. *b*, optical absorption spectra of isolated complexes: *dashed lines*, ascorbate-reduced samples; *solid lines*, dithionite-reduced samples. *c*, X-band CW-EPR spectra of reduced 2Fe-2S clusters present in samples of isolated complexes. Spectra were recorded at 20 K, 1.7 mT modulation amplitude, and 2.015 milliwatts of microwave power. *d*, photosynthetic growth under anaerobic conditions at pH 7. MTR*bc*_1_ (*R. capsulatus* strain lacking cytochrome *bc*_1_ operon) was used as negative control. *e*, heterotrophic growth in aerobic dark conditions (cytochrome *bc*_1_-independent) at pH 7 was used as positive control.

The mutated complexes contained all three catalytic subunits, as indicated by the SDS electrophoretic profiles (Fig. 3*a*). Optical (UV-visible), and electron paramagnetic resonance spectroscopy (EPR) showed that the mutants contained all redox active cofactors: heme *c*_1_, hemes *b* (*b*_L_ and *b*_H_) and 2Fe-2S cluster (Figs. 2*b* and 3, *b* and *c*). Although the spectral and redox properties of 2Fe-2S and heme *c*_1_ remained unchanged in the mutants, the properties of hemes *b* were modified. We emphasize the results of EPR analysis (Fig. 2*b*), which in this case is most informative because, unlike UV-visible spectroscopy, it allows for a complete spectral separation of *g* transitions originating from each of the hemes *b*: in native enzyme *g* = 3.78 and 3.44, corresponding to *g*_z_ transition of heme *b*_L_ and heme *b*_H_, respectively (31).

The mutationally imposed changes in ligation pattern resulted in the disappearance of the *g*_z_ transition of the targeted heme at the position characteristic for a native enzyme with concomitant appearance of new transitions at *g* = 3.22 and 3.15 (Fig. 2*b*, *black*). More specifically, these new transitions replaced *g* = 3.78 in H198K, *g* = 3.44 in H212K, and both *g* = 3.78 and 3.44 in the double mutant H212K/H198K. In general, these shifts reflect a lowering of the symmetry and changes from a highly axial to a more rhombic low spin heme (32). The new transitions *g* = 3.22 and 3.15 are assigned to *g*_z_ transitions of low spin hemes *b* coordinated by His-Lys. Support for this assignment comes from the differential effect of antimycin, an inhibitor that binds to the Q_i_ site in the proximity of heme *b*_H_, and is known to affect the EPR spectrum of heme *b*_H_ (33). Antimycin clearly affects the *g* = 3.22 and 3.15 of heme *b*_H_ in H212K and H212K/H198K, whereas in H198K it has a weak or no effect (Fig. 2*b*, *gray*). In H212K/H198K, for which both hemes *b*_L_ and *b*_H_ contribute to 3.22 and 3.15 transitions, the effect of antimycin is intermediary between the largest and smallest effect seen in H212K and H198K, respectively.

##### His-Lys Coordinated Hemes b Have Markedly Elevated E_m_, Effectively Modulating ΔE_m__b

**FIGURE 4.**
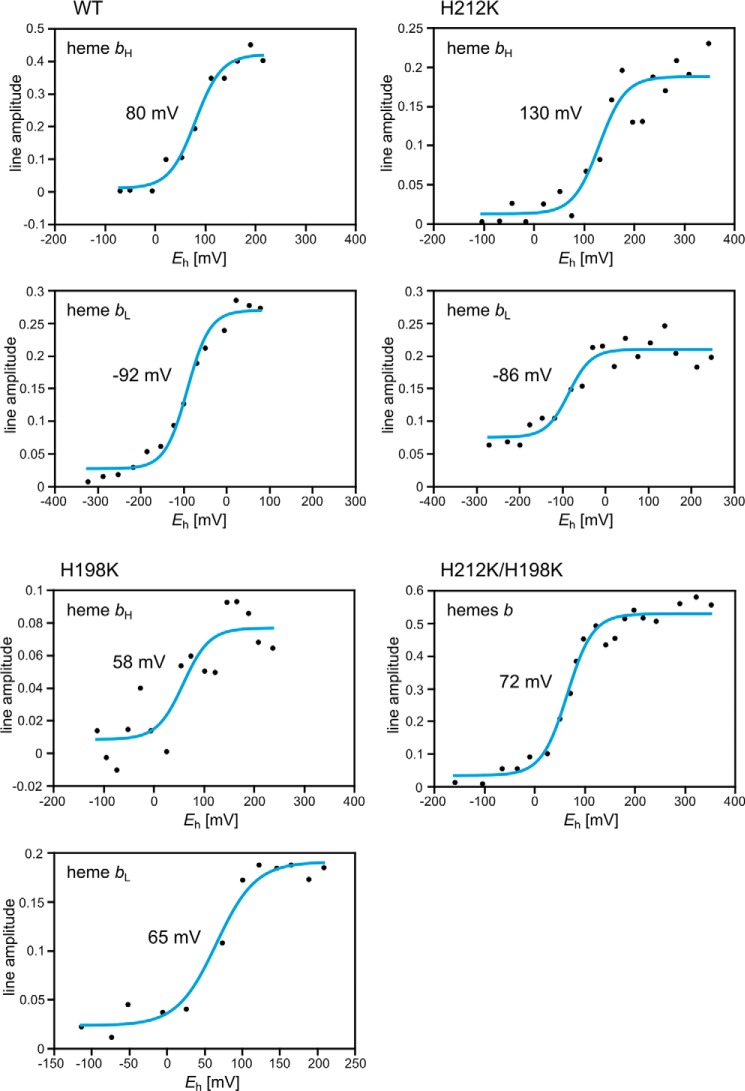
**Midpoint potentials of hemes *b* determined via EPR-monitored redox titrations.** Each *blue line* represents the Nernst titration curve. The respective *E*_m_ values are given in the middle of each plot. Titrations were performed on isolated chromatophores at pH 7.0. Amplitudes of heme *b*_L_ and heme *b*_H_ were monitored at respective *g* values: 3.78 and 3.44 in WT, 3.22 and 3.44 in H198K, 3.78 and 3.2 in H212K, and 3.2 in H212K/H198K.

Dark equilibrium redox titrations revealed that changing the ligation pattern from bis-His to His-Lys in hemes *b* leads to a significant increase in their *E*_m_ values. This increase reaches 50 mV for heme *b*_H_ in H212K and almost 160 mV for heme *b*_L_ in H198K (Fig. 4 and Table 1). At the same time, there was no significant change of *E*_m_ (only ∼10 mV increase was observed in *E*_m_ for heme *b*_L_ in H212K or 20 mV decrease of *E*_m_ for heme *b*_H_ in H198K) in the heme that was not subject to modification in these mutants. A large increase in the *E*_m_ of just one heme (*b*_L_ or *b*_H_) effectively modulated Δ*E*_m__b in those mutants (Fig. 2*a*). In H212K Δ*E*_m__b was increased, and *E*_m_ of heme *b*_H_ rose above the *E*_m_ of quinone pool in the membrane (34, 35). In H198K Δ*E*_m__b was decreased to the point that both hemes became almost isopotential. In H212K/H198K, both His-Lys ligated hemes showed *E*_m_ of ∼72 mV. This means that only heme *b*_L_ elevated its *E*_m_. Consequently, the Δ*E*_m__b in this mutant is similar to that of H198K.

**TABLE 1:** **Selected properties of wild-type and Lys mutants**

Form of *bc*_1_	Phenotype	*E*_m7_		EPR *g*_z_ value		Rates of hemes *b* reduction			Steady-state activity
		*b*_L_	*b*_H_	*b*_L_	*b*_H_ (+antimycin)	pH 6	pH 7	pH 9	
		*mV*				*s*^−*1*^			*s*^−*1*^
WT	Ps^+^	−92	80	3.78	3.44 (3.47)	566	1051	1791	149 ± 7
H212K	Ps^+^	−86	130	3.78	3.22/3.15 (3.26)	479	822		121 ± 6
H198K	Ps^+^	65	58	3.22	3.44 (3.47)	392	770	1506	118 ± 5
H212K/H198K	Ps^+^	∼72		∼3.2	∼3.2 (∼3.24)	420	706	1548	91 ± 6

##### Mutants with Large Changes in ΔE_m__b Are Functional in Vitro and in Vivo

Remarkably, all mutants showed the capability to grow under cytochrome *bc*_1_-dependent photoheterotrophic conditions (*i.e.* exhibited the Ps^+^ phenotype), which indicated that mutated cytochromes *bc*_1_ are functional *in vivo* (Table 1 and Fig. 3, *d* and *e*). The functional competence of these mutants was confirmed by light-induced electron transfer measurements that allow monitoring of individual reactions associated with the catalytic cycle. In brief, these reactions include oxidation of quinol at the Q_o_ site, which delivers one electron to one cofactor chain (the c chain) consisting of a 2Fe-2S cluster, heme *c*_1_ and heme *c*_2_. The other electron is used to reduce heme *b*_L_ in a separate chain (the b chain), followed by cross-membrane electron transfer to heme *b*_H_, which then reduces the occupant of the Q_i_ site.

**FIGURE 5.**
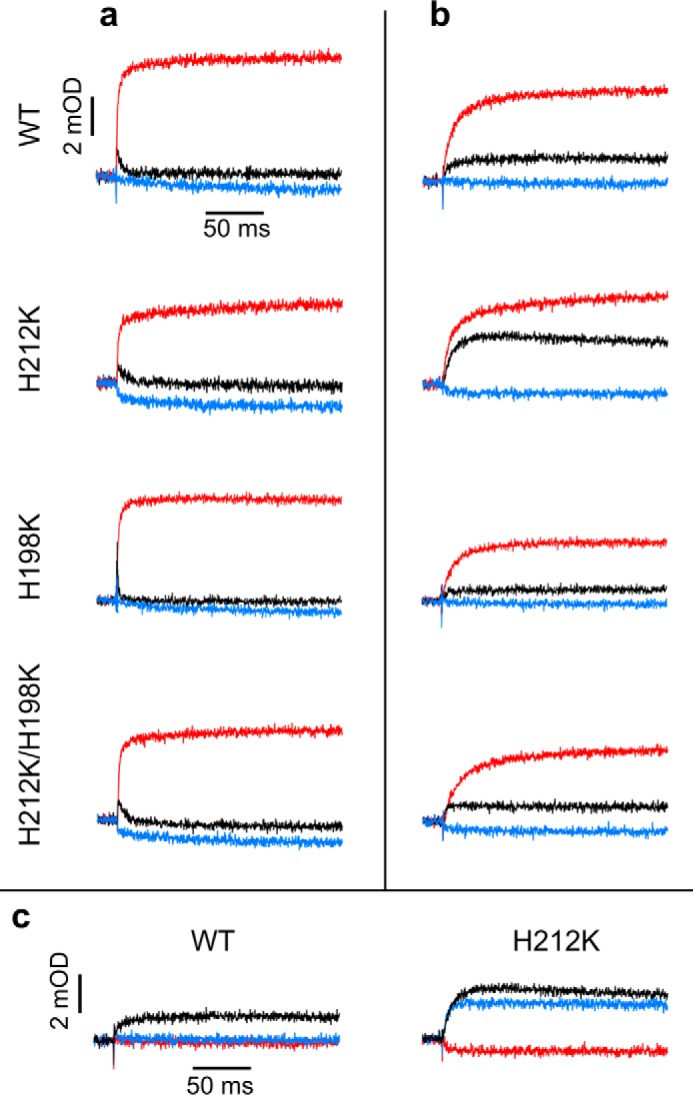
**Single flash-activated transients reveal fast heme *b* reduction and changes in equilibria of partial reactions in Lys mutants.** *a*, transients in the absence of inhibitor (*black*) after addition of antimycin (*red*) and subsequent addition of myxothiazol (*blue*) recorded at pH 7 and ambient potential of 100 mV (Q pool half-reduced). *b*, same as in *a*, except that ambient potential was 200 mV (Q pool oxidized). *c*, same as in *b*, except that the order of addition of inhibitors was inverted: antimycin (*red*) was subsequent to myxothiazol (*blue*). Transient hemes *b* reduction kinetics for WT and H198K were followed at 560–570 nm and for H212K and H212K/H198K at 557–570 nm.

The kinetic transients shown in Fig. 5 monitored changes in the oxidation state of heme *b*_H_ associated with electron transfer through the b chain. When the quinone (Q) pool is poised half-reduced before light activation (ambient potential of 100 mV at pH 7) (Fig. 5*a*), cytochrome *bc*_1_ in native chromatophores and in the absence of any inhibitors displays a fast reduction of heme *b*_H_ followed by its fast reoxidation (WT, *black trace*). The reduction phase is mediated by an electron from the Q_o_ site, whereas the reoxidation phase results from electron transfer to the occupant of the Q_i_ site. In the presence of antimycin, the reoxidation phase is blocked, and heme *b*_H_ remains reduced within the time domain monitored in the experiment (WT, *red trace*). The rate of this reduction (measured at pH 7), as well as the rates measured at two other pH values (pH 6 and 9, which test conditions of different driving force provided by substrates) are listed in Table 1. All phases of the electron transfer described above and involving heme *b*_H_ (in the absence of any inhibitors and in the presence of antimycin) are observed in all Lys mutants, and the measured rates of heme *b*_H_ reduction at pH 6, 7, or 9 are only slightly lower than the corresponding rates in wild type (Fig. 5*a* and Table 1). This reveals a full competence of catalytic reactions of Q_o_ and Q_i_ sites in the mutants. We note the smaller amplitude of heme *b*_H_ reduction in the presence of antimycin in H212K mutant. This is an effect of a partial prereduction of this heme before activation, which is a consequence of an elevated *E*_m_ exceeding the *E*_m_ of the Q pool.

**FIGURE 6.**
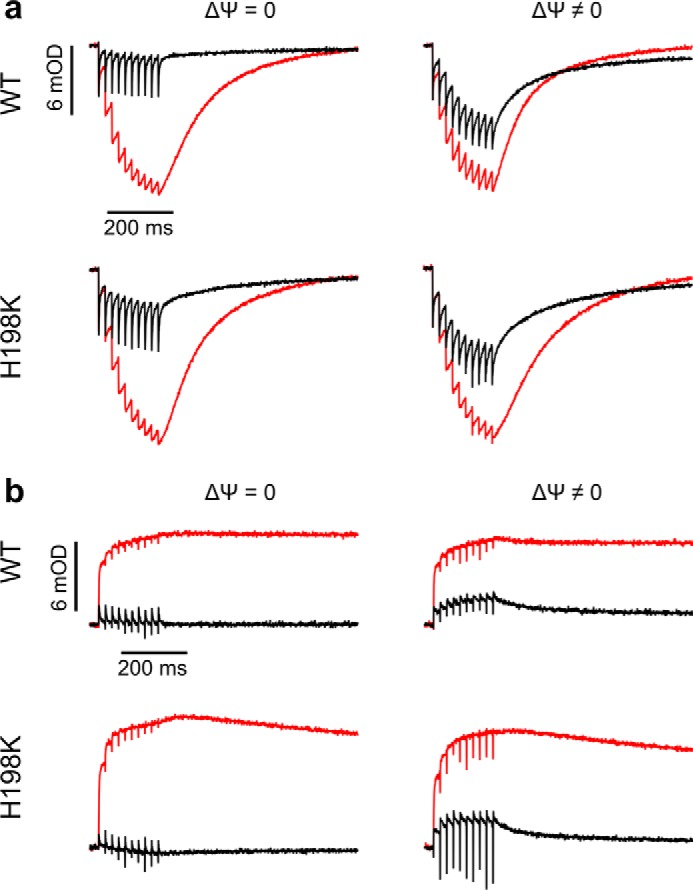
**Multiple flash-activated competence of enzyme with isopotential hemes *b* in the absence and presence of electric membrane potential.** *a*, transients for hemes *c* oxidation and rereduction in WT and H198K activated by 10 flashes separated by 20 ms in the absence of inhibitors (*black*) and in the presence of antimycin (*red*), in the absence (ΔΨ = 0) or presence (ΔΨ ≠ 0) of the membrane potential, recorded at pH 7 and ambient potential of 100 mV. *b*, transients for hemes *b* recorded as in *a*.

Kinetic competence of the mutants was also evident in the response of cytochrome *bc*_1_ to multiple flash activation in both the absence and presence of membrane potential. In the absence of inhibitors, hemes *c* in native enzyme and H198K undergo several cycles of fast oxidation and rereduction (Fig. 6*a*, *black*), and hemes *b* undergo several cycles of fast reduction and reoxidation (Fig. 6*b*, *black*). As expected, antimycin impedes the rereduction phase of hemes *c* (Fig. 6*a*, *red*) and blocks the reoxidation phase of hemes *b* (Fig. 6*b*, *red*). The membrane potential diminishes the effectiveness of the rereduction of hemes *c* and reoxidation of hemes *b*, leading to partial accumulation of oxidized hemes *c* and reduced hemes *b*, but the magnitude of this effect in native enzyme and H198K is similar. In multiple flash experiments, H212K behaved similarly to H198K.

Enzymatic competence of the Lys mutants is evident in the measured steady-state turnover rates, which remain in good correlation with the results of light-induced electron transfer measurements. As listed in Table 1, the turnover rates in H212K or H198K single mutants and in H212K/H198K double mutant decrease by 20 and 40%, respectively, compared with WT. Clearly, all Lys mutants retain significant enzymatic activity.

##### Changes in ΔE_m__b Affect Equilibria of Partial Reactions

Kinetic traces measured when the Q pool is fully oxidized before activation (ambient potential of 200 mV at pH 7) are compared in Fig. 5*b*. Under these conditions, the amount of quinol molecules after activation is limited, and consequently, approximately only one quinol is oxidized in every Q_o_ site. It follows that a difference between the level of reduction of heme *b*_H_ in the absence and presence of antimycin reports equilibration of electron between heme *b*_H_^3+^/*b*_H_^2+^ and quinone/semiquinone (Q/SQ) couples at the Q_i_ site. In native enzyme and H198K mutant, this difference is large, indicating that the electron resides mostly on semiquinone at the Q_i_ site (∼80%). However, in H212K mutant, the amplitude of reduced heme *b*_H_ in the absence of inhibitors is significantly larger than in wild type. This indicates that equilibrium between heme *b*_H_^3+^/*b*_H_^2+^ and Q/SQ is shifted so that the electron resides mostly on heme *b*_H_ (at the expense of semiquinone). This shift is a consequence of an elevated *E*_m_ of heme *b*_H_ in H212K, which exceeds the *E*_m_ of Q/SQ at pH 7 (Fig. 2*a*).

Another way to monitor electron equilibration between heme *b*_H_ and the occupant of the Q_i_ site benefits from the occurrence of reverse reaction in the Q_i_ site (Fig. 5*c*). When the Q pool is fully oxidized before light activation, and the Q_o_ site is blocked by an inhibitor (myxothiazol), light-induced reduction of heme *b*_H_ reports electron transfer from quinol that entered the Q_i_ site to heme *b*_H_. However, in a native enzyme, this reaction cannot be observed at pH 7 (Fig. 5*c*, *blue*), which seems consistent with the fact that *E*_m_ of heme *b*_H_ is lower than *E*_m_ of the Q pool. On the other hand, at this pH, the reverse electron transfer from Q_i_ site quinol to heme *b*_H_ is prominent in H212K (Fig. 5*c*, *blue*). Furthermore, the amplitude of heme *b*_H_ reduction in this reaction (*i.e.* in the presence of myxothiazol) is almost as large as the amplitude of heme *b*_H_ reduction in the absence of any inhibitor.

Equilibration of electrons between heme *b*_H_ and the occupant of the Q_i_ site in H212K mutant under the conditions described in Fig. 5 (*b* and *c*) allowed us to estimate the *E*_m_ values for the SQ/QH_2_ and Q/SQ redox couples at the Q_i_ site at pH 7. The extent of forward reaction (electron transfer from heme *b*_H_ to Q) monitored in Fig. 5*b* will reflect the difference between the *E*_m_ of heme *b*_H_^3+^/*b*_H_^2+^ and the *E*_m_ of Q/SQ couples, whereas the extent of the reverse reaction (electron transfer from quinol to heme *b*_H_) monitored in Fig. 5*c* will reflect the difference between the *E*_m_ of heme *b*_H_^3+^/*b*_H_^2+^ and the *E*_m_ of SQ/QH_2_ couples. Based on these assumptions, and considering *E*_m7_ = 130 mV for heme *b*_H_ in an H212K mutant, we estimate values of *E*_m7_ for the Q/SQ couple to be ∼114 mV, and *E*_m7_ for the SQ/QH_2_ couple to be 147 mV. The average redox midpoint potential for Q/QH_2_ (*E*_m7_ for Q/QH_2_) is then 130 mV, which makes it isopotential with the *b*_H_^3+^/*b*_H_^2+^ couple in an H212K mutant and ∼30 mV higher than the *E*_m_ of Q in the Q pool reported in the literature. The split in quinone redox couples defines the stability constant for SQ (35, 36); thus, our estimates of *E*_m7_ for Q/SQ and *E*_m7_ for SQ/QH_2_ indicate the stability constant (log(*K*_s_) = [*E*_m(Q/SQ)_ − *E*_m(SQ/QH2)_]/60) for SQ_i_ at the level of 3 × 10^−1^.

## Discussion

We have examined the effect of large changes in ΔG on electron transfer between hemes *b* in cytochrome *bc*_1_. These changes were implemented by significant increases in the *E*_m_ values of the hemes, which came as a result of mutating the native bis-His coordination of the heme iron into the His-Lys coordination. Our results indicate that the natural difference in *E*_m_ values of the two hemes *b* (Δ*E*_m__b) of 170 mV can be increased to 210 mV (in H212K) or diminished to almost 0 (in H198K and double mutant H212K/H198K), and the complex still remains functional *in vivo*, retaining the catalytically relevant electron transfer from the Q_o_ to Q_i_ sites measured *in vitro* under the absence or presence of membrane potential. The electron flow through cofactor chains of cytochrome *bc*_1_ in all those mutants proceeded from the primary electron donor (QH_2_ in the Q_o_ site) to the final electron acceptor (Q or SQ in the Q_i_ site) at physiologically and mechanistically competent rates, although changes in Δ*E*_m__b affected equilibrium levels of partial reactions in the coupled electron transfer chains. Clearly, these changes did not introduce any significant barrier to electron transfer from donor to final acceptor. It thus appears that cytochrome *bc*_1_ can accommodate large changes in Δ*E*_m__b without hampering catalysis, as long as these changes do not impose overly endergonic steps on the route of electron transfer from substrate to product. The previously reported moderate decrease in *E*_m_ for heme *b*_H_ in yeast cytochrome *bc*_1_ falls into this category of changes, *i.e.* ones not imposing overly endergonic steps, and thus, consistent with our observations, did not affect significantly the measured turnover rate of the enzyme (20).

Considering the direction of electron flow and the direction of the gradient of *E*_m_ in relation to the vector of the electric membrane potential, four configurations are possible (Fig. 1). In cytochrome *bc*_1_ (11, 12), cytochrome *b*_6_*f* (37, 38), nitrate reductase A (39, 40), and quinol-fumarate reductase (41, 42), the high potential heme *b* is located at the negative side of the membrane (Fig. 1*a*), which at first would seem to be in line with the concept of the obligatory difference in *E*_m_ to overcome membrane potential. However, in formate dehydrogenase N (40, 43, 44) and probably in membrane-bound [Ni-Fe] hydrogenase (45, 46), the low potential heme is located at the negative side of the membrane, and the electron is transferred against both the electric membrane and the redox potential of hemes *b* (Fig. 1*b*), which remains at odds with the concept presented above. The third possibility found in succinate-quinone reductase (47–50), NADPH oxidase (51), or cytochrome b_561_ family (52–54) is simply an inversion of the case in Fig. 1*a* with electron transfer from high to low potential facilitated by the presence of electric membrane potential (Fig. 1*c*). The fourth possibility (Fig. 1*d*) perhaps concerns proton motive force-driven electron transfer in thiosulfate reductase, if heme *b*_L_ in this enzyme is located close to the menaquinone binding site, as in formate dehydrogenase N (which seems likely, based on sequence similarities between the cytochrome *b* subunits of these complexes) (55).

The occurrence of all these configurations suggests that there is no a universal rule for the arrangement of *E*_m_ values of hemes *b* among various transmembrane cytochromes *b*. This, however, becomes understandable in light of our observation that large Δ*E*_m__b and fine tuning of *E*_m_ values of hemes *b* of cytochrome *bc*_1_ are not required for efficient cross-membrane electron transfer. Another example of tolerance for changes in Δ*E*_m__b for cross-membrane electron transfer concerns *Bacillus subtilis* succinate-quinone reductase. In this case, the mutant with the His-Met ligated heme *b*_L_ having the *E*_m_ increased by at least 100 mV is expected to diminish the barrier for the uphill step of electron transfer from heme *b*_H_ to heme *b*_L_ (Fig. 1*c*) but, at the same time, to introduce a barrier for electron transfer from heme *b*_L_ to menaquinone. However, such modification, despite lowered enzymatic activity, did not eliminate the function of the enzyme *in vivo*. Furthermore, its activity to reduce ubiquinone was not significantly affected (48).

**FIGURE 7.**
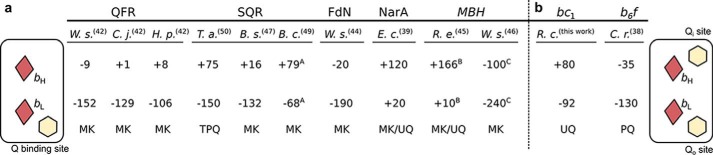
**Localization of low and high potential hemes *b* with respect to the quinone binding site in cytochrome *b* subunits of enzymes involved in cross-membrane electron transfer.** *a*, in cytochromes *b* with one Q binding site, low potential heme (*b*_L_) is adjacent to the Q binding site, whereas high potential heme (*b*_H_) faces the opposite side of the membrane. *b*, in cytochromes *bc*, the cytochrome *b* subunit contains two Q binding sites and heme *b*_L_ is adjacent to one site (Q_o_), whereas heme *b*_H_ is adjacent to the other (Q_i_). *Brown diamonds*, hemes *b*; *yellow hexagons*, Q binding sites. *Numbers* indicate *E*_m_ values (in mV) for pH 7. *Superscripts A*, *B*, and *C* refer to *E*_m_ values for pH 7.6, 7.2, and 7.5, respectively. Types of natively used quinones are given below the *E*_m_ values. Localization of hemes in membrane-bound [Ni-Fe] hydrogenase (*MBH*) is presumable; thus, its name is given in italic. Protein names abbreviations as in Fig. 1. Other abbreviations as follow: *B. c., Bacillus cereus; B. s., Bacillus subtilis; C. j., Campylobacter jejuni; C. r., Chlamydomonas reinhardtii; E. c., Escherichia coli; H. p., Helicobacter pylori; R. e., Ralstonia eutropha; R. c., Rhodobacter capsulatus; T. a., Thermoplasma acidophilum; W. s., Wolinella succinogenes;* MK, menaquinone; TPQ, thermoplasmaquinone; PQ, plastoquinone; UQ, ubiquinone.

Extrapolating all these observations to other cytochromes *b*, it can be proposed that, in all those proteins, hemes *b* simply act as electronic connectors for the catalytic sites with no fine tuning in Δ*E*_m__b required for efficient electron transfer. It follows that the existence of Δ*E*_m__b in transmembrane embedded hemes *b* is not an element in the control of electron flow across the membrane. Rather, it may be a consequence of a higher probability of coexistence of two cofactors having different *E*_m_ in comparison to the case when the two cofactors have similar *E*_m_. Intriguingly, we found one resemblance for enzymes with one quinone binding site: from two hemes *b* of different potentials, the one with the lower potential is adjacent to the quinone binding site (Fig. 7) (38, 39, 42, 44–47, 49, 50). Further studies are required to verify whether this has any functional relevance or is just a consequence of structural constraints. Nevertheless, this resemblance may be useful in predicting the location of low and high potential heme *b* in quinol-binding cytochromes, for which such assignments are yet to be made.

The demonstrated robustness of cytochrome *b* to changes in ligation pattern and associated changes in Δ*E*_m__b raises an interesting question as to whether variation in the heme ligation patterns exists in natural membrane proteins of similar design and/or function. Such variation is evident in the group of cytochromes *c* for which bis-His (4, 56, 57), His-Met (4, 56, 57), His-Lys (58), His-Cys (59–61), or even His-Tyr (62, 63) patterns ligating heme iron are observed. However, in most known cytochromes *c*, the heme binding domains are water-soluble or solvent-exposed (so far, the heme *c*_x_ from cytochrome *b*_6_ is the only known exception (5, 64, 65)), whereas in cytochromes *b* these domains can be located either in membrane or in aqueous phase (5). It may seem that the location of the heme binding motifs outside the membrane environment is one of the factors increasing structural flexibility to accommodate diverse heme ligation. Indeed, all rare cases of His-Met (66, 67), Lys-Met (68), or bis-Met (69, 70) ligations in cytochromes *b* are relevant only to water-soluble domains. In fact, to our knowledge, no cases have been reported so far of Met or Lys serving naturally as axial ligands for hemes bound within the integral membrane proteins.

However, our Lys mutants prove that His-Lys ligation for hemes *b* can occur within the transmembrane helix bundle of cytochrome *b*, yielding functional hemes that contain a low spin form of iron ion. Notably, the H212K mutant was originally isolated as a reversion for H212N, the so-called heme *b*_H_ knock-out, with impaired electron transfer at the level of the heme *b*_H_/Q_i_ site. Likewise, the His-Met heme *b* mutant of *B. subtilis* succinate-quinone reductase was isolated as a reversion of nonfunctional Leu mutant (48). This all indicates that an assembly of functionally active low spin heme *b* present within the transmembrane segment of the protein and coordinated by His and Lys or Met is feasible from a protein engineering perspective. If this is the case, one should expect that there are natural cases of His-Lys and His-Met ligation patterns for membranous heme proteins (71) that still await identification, especially because the range of scrutinized heme proteins is currently continuously widening.

## Author Contributions

S. P. performed most of the biochemical and spectroscopic experiments and analyzed data; P. K. performed light-induced electron transfer measurements; E. C. constructed mutants and contributed preliminary results; A. B. performed enzymatic activity assays; and S. P., M. S., and A. O. designed the experiments, interpreted the data, and cowrote the paper. All authors reviewed the results and approved the final version of the manuscript.

## Footnotes

2 The abbreviations used are:

Q_o_: quinol oxidation site
Q_i_: quinone reduction site
EPR: electron paramagnetic resonance spectroscopy
Q: quinone
QH_2_: quinol
SQ: semiquinone.
